# LncRNA DLEU1 as a novel therapeutic target and prognostic biomarker in various malignancies: a meta-analysis, bioinformatics analysis and systematic review

**DOI:** 10.3389/fonc.2026.1805194

**Published:** 2026-04-13

**Authors:** Shaopu Hu, Yanru Cai, Qian Yang

**Affiliations:** 1 The First Affiliated Hospital, Hebei University of Chinese Medicine, Shijiazhuang, China; 2 Hebei Key Laboratory of Turbidity Toxin Syndrome, Hebei University of Chinese Medicine, Shijiazhuang, China; 3 Key Laboratory of Integrated Chinese and Western Medicine for Gastroenterology Research (Hebei), Shijiazhuang, China

**Keywords:** bioinformatics, biomarker, cancer, LncRNA DLEU1, meta-analysis

## Abstract

**Background:**

Accumulating evidence indicates that the aberrantly expressed long non-coding RNA DLEU1 may serve as a potential therapeutic target and prognostic biomarker in various cancers; however, these findings remain inconclusive. This study systematically evaluated the prognostic value of DLEU1 via a meta-analysis, while also summarizing its biological functions and underlying mechanisms in malignancies.

**Methods:**

A systematic literature search was performed in PubMed, Web of Science, Embase, and the Cochrane Library from inception until October 10, 2025. Pooled hazard ratios or odds ratios with 95% confidence intervals were calculated to assess the associations between DLEU1 expression and patient survival or clinicopathological parameters. These findings were subsequently validated using the GEPIA2 platform. Potential target genes of DLEU1 were predicted via the StarBase database, followed by Gene Ontology and Kyoto Encyclopedia of Genes and Genomes pathway enrichment analyses. Additionally, we systematically reviewed and summarized the reported functional roles and molecular mechanisms of DLEU1 over the past five years.

**Results:**

This meta-analysis, incorporating nine studies with a total of 938 cancer patients, demonstrated that elevated DLEU1 expression was significantly associated with shortened overall survival and adverse clinicopathological features, including lymph node metastasis and advanced TNM stage. In contrast, no significant correlations were observed with age, gender, tumor size, or tumor differentiation. Independent validation via the GEPIA2 database confirmed DLEU1 upregulation in six cancer types and its correlation with poorer survival. Furthermore, a total of 254 potential target genes of DLEU1 were identified through StarBase, and these target genes were significantly enriched in various cancer-related pathways, such as Rap1 signaling pathway, cAMP signaling pathway, PI3K-Akt signaling pathway, apoptosis, and calcium signaling pathway.

**Conclusion:**

Upregulated expression of lncRNA DLEU1 is associated with adverse clinicopathological features and poor prognosis in cancer patients, establishing it as a promising prognostic biomarker and therapeutic target across multiple malignancies.

**Systematic Review Registration:**

https://www.crd.york.ac.uk/PROSPERO/, identifier CRD420251167807.

## Introduction

Cancer remains a major cause of death and a serious global health challenge (1). According to the 2022 Global Cancer Statistics, there were approximately 20 million new cancer cases and 9.7 million cancer-related deaths worldwide in 2022. The number of new cases of cancer is predicted to reach 35 million by 2050 (2). Although considerable progress has been made in various treatment modalities including molecular targeted therapy and immunotherapy, clinical benefits for patients remain limited, and the prognosis for most patients with malignant tumors remains unfavorable. The lack of effective biomarkers for early prognosis prediction is one of the main reasons for this, which often results in most tumor patients being diagnosed at advanced stages (3). Therefore, developing novel therapeutic targets and prognostic biomarkers is of significant clinical value.

Long non-coding RNAs (lncRNAs), a class of non-coding RNA molecules exceeding 200 nucleotides in length, were once regarded as transcriptional “noise” generated by RNA polymerase II and thought to be devoid of biological function (4). Nevertheless, accumulating evidence confirms that aberrantly expressed lncRNAs play key roles in tumorigenesis, functioning either as tumor suppressors or oncogenes to regulate critical cellular processes such as proliferation, migration, and apoptosis (5). Moreover, their significant correlation with clinicopathological features and patient prognosis highlights their potential as emerging targets for cancer therapy and prognostic assessment (6, 7). With the rapid development of high-throughput technologies, numerous functionally relevant lncRNAs have been identified and are now under extensive investigation.

LncRNA deleted in lymphocytic leukemia 1 (DLEU1) is located on chromosome 13q14.3, a region which is frequently deleted in hematopoietic malignancies, and was initially proposed to function as a potential tumor suppressor gene. However, recent studies have demonstrated that DLEU1 is overexpressed in various malignancies and functions as an oncogene to regulate tumor malignant phenotypes (8, 9). Mechanistically, DLEU1 contributes to tumorigenesis and progression by acting as a competitive endogenous RNA (ceRNA) that sponges microRNAs (miRNAs), thereby modulating gene expression. For instance, DLEU1 is upregulated in colorectal cancer tissues and cell lines, and its knockdown suppresses cell proliferation and migration via the miR-320b/PRPS1 axis (10). Similarly, DLEU1 modulates malignant biological behaviors in ovarian cancer (11), cholangiocarcinoma (12), and oral squamous cell carcinoma (13) through interactions with various miRNAs, underscoring its potential as a therapeutic target across multiple cancer types.

As a potential tumor biomarker, DLEU1 has garnered significant attention for its prognostic value in cancer. Multiple studies have reported that high DLEU1 expression is strongly associated with poor prognosis across various cancers, including cervical cancer (14, 15), pancreatic ductal adenocarcinoma (16), nasopharyngeal carcinoma (17), gastric cancer (18), breast cancer (19), non-small cell lung cancer (20), osteosarcoma (21) and hepatocellular carcinoma (22). However, due to limitations such as small sample sizes in individual studies, its prognostic significance remains controversial. Therefore, we systematically searched relevant studies and conducted this meta-analysis to provide an evidence-based assessment of the prognostic role of DLEU1 in human cancers. Additionally, we summarize the molecular mechanisms associated with DLEU1 to offer insights for future basic and translational research.

## Materials and methods

The present meta-analysis was conducted in accordance with the PRISMA guidelines (https://www.prisma-statement.org/) and was registered on the PROSPER database (https://www.crd.york.ac.uk/PROSPERO/) under the registration number CRD420251167807.

### Literature search strategy

Two investigators (Shaopu Hu and Yanru Cai) independently performed a systematic literature search of four electronic databases: PubMed, Embase, Web of Science, and the Cochrane Library. The search aimed to identify studies evaluating the correlation between DLEU1 expression levels and clinicopathological parameters or prognosis in cancer patients. The terms of ((((DLEU1) OR (LncRNA DLEU1)) OR (Long non coding RNA DLEU1)) OR (Long non-coding RNA DLEU1)) AND ((((((Cancer) OR (Carcinoma)) OR (Tumor)) OR (Tumour)) OR (Neoplasm)) OR (Malignancy)) were used as the search strategy. The search period spanned from the inception of each database to October 10, 2025, with no restrictions on language.

### Inclusion and exclusion criteria

Inclusion criteria were defined based on the PICOS principle:

1. Population: patients with various histopathologically confirmed malignancies;
2. Intervention: high expression of DLEU1 in cancer tissues, as detected by methods such as qRT-PCR;
3. Comparison: low expression of DLEU1 in cancer tissues from the same cohort;
4. Outcomes: overall survival (OS) and clinicopathological parameters (e.g., TNM stage, lymph node metastasis). Hazard ratios (HRs) with 95% confidence intervals (CIs) for survival were either directly reported or estimated from Kaplan-Meier curves;
5. Study design: prospective or retrospective cohort studies.

Exclusion criteria were as follows:

1. studies that analyzed a panel of lncRNAs collectively rather than evaluating DLEU1 individually.
2. publications such as editorials, letters, case reports, or retracted articles.
3. studies from which essential data were unavailable.
4. studies where data on patient prognosis or clinicopathological characteristics were solely derived from bioinformatics analysis platforms without experimental validation.

### Data extraction and quality assessment

Two investigators (Shaopu Hu and Yanru Cai) independently extracted the following data from each eligible study: first author, publication year, country, sample size, cancer type, outcome measures, method of DLEU1 detection, follow-up duration in months, and hazard ratios for survival outcomes with corresponding 95% CIs. The quality of the included studies was assessed using the Newcastle-Ottawa Scale (NOS, https://www.ohri.ca//programs/clinical_epidemiology/oxford.asp) (23). Studies were classified as high quality (NOS ≥7), moderate quality (NOS 4-6), or low quality (NOS ≤3) (24). Any discrepancies encountered during data extraction or quality assessment were resolved through consensus discussion with a third investigator (Qian Yang).

### Statistical analysis

The statistical analyses in this meta-analysis were performed following methodologies established in our previous research (3). Briefly, pooled effect sizes and forest plots were generated using Review Manager (RevMan) version 5.3. For studies presenting only Kaplan-Meier survival curves, the Engauge Digitizer 10.0 software was utilized to extract data and estimate HRs with 95% CIs. The associations between DLEU1 expression levels and clinicopathological parameters (including age, gender, differentiation, lymph node metastasis, tumor size, and TNM stage) in cancer patients were evaluated using odds ratios (ORs) with 95% CIs. Heterogeneity among the included studies was assessed using the Cochran’s Q test and the I² statistic. A fixed-effects model was applied when no significant heterogeneity was observed (I² < 50%, p > 0.1); otherwise, a random-effects model was employed (I² ≥ 50%, p ≤ 0.1). Given that all studies included in this analysis were observational and inherently possess significant heterogeneity, to enhance the robustness and reliability of the results, all analyses were conducted using a random-effects model for statistical evaluation. Potential publication bias was examined through visual inspection of funnel plots. Sensitivity analysis was performed by sequentially omitting one study at a time to evaluate the influence of each individual study on the pooled HR for OS.

### Bioinformatic analysis of DLEU1

The expression levels and survival analysis of DLEU1 across multiple malignancies was conducted using the Gene Expression Profiling Interactive Analysis 2 (GEPIA2) database (http://gepia2.cancer-pku.cn/) (25). Potential target genes of DLEU1 were identified through the StarBase platform (http://starbase.sysu.edu.cn/) (26). Subsequently, these predicted target genes were subsequently analyzed for functional enrichment using Gene Ontology (GO) and the Kyoto Encyclopedia of Genes and Genomes (KEGG) pathway databases through an online bioinformatics platform (https://www.bioinformatics.com.cn/) (27). The significantly enriched terms and pathways were thereby identified and presented in visual formats.

## Results

### Literature screening

**Figure 1:**
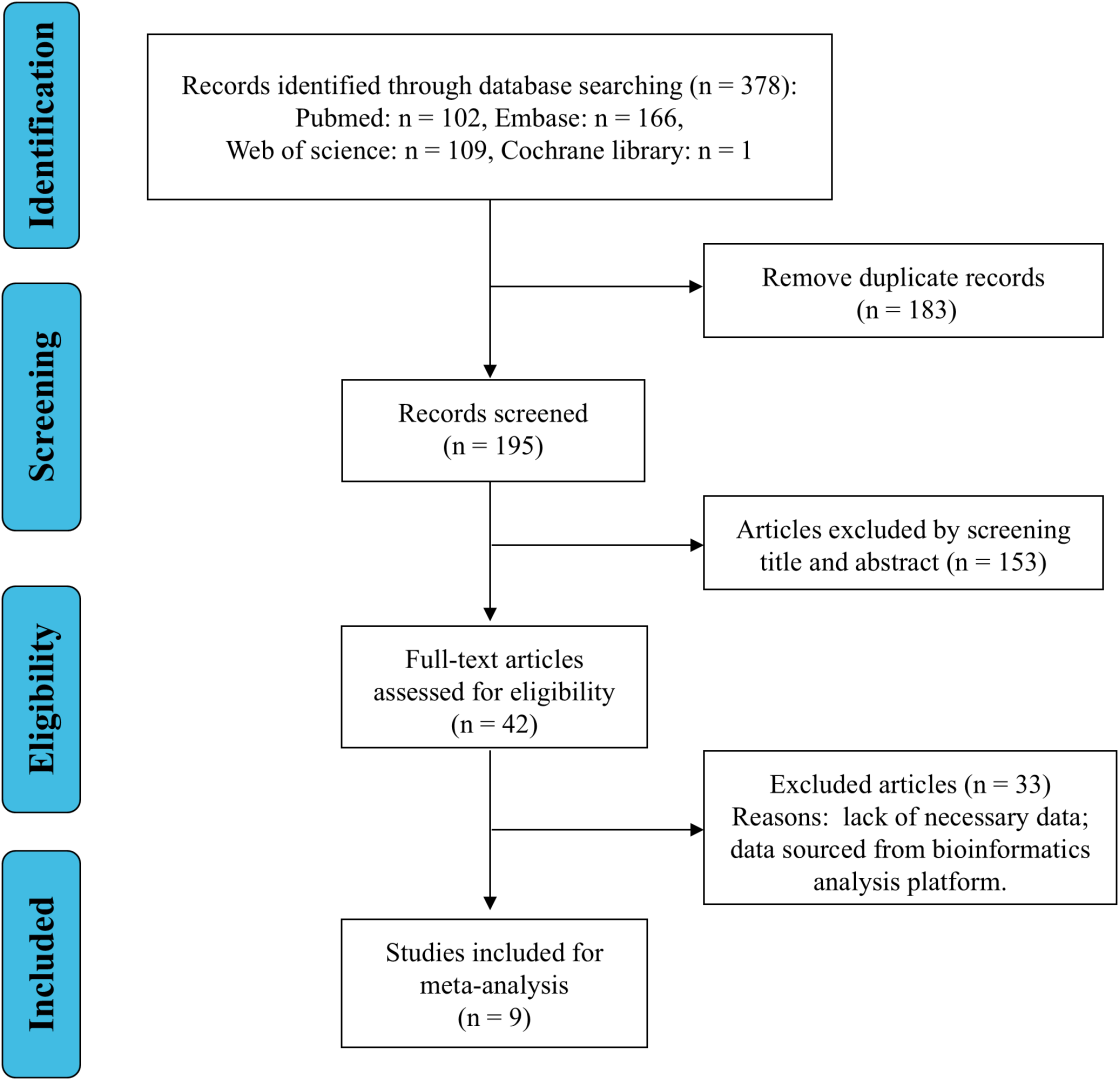
Flowchart of literature search and screening.

The process of literature search and screening for this study is shown in **Figure 1**. Based on the established search strategy, a total of 378 articles were initially identified from four databases (Pubmed=102, Embase=166, Web of science=109, Cochrane library=1), among which 183 duplicate articles were eliminated. After reviewing the titles and abstracts of each article, 153 articles were further deleted as they were not related to tumors or belonged to types such as reviews, letters, case reports, or retracted article. Through a detailed reading of the full texts of the remaining 42 articles, 33 studies were further excluded as they lacked data for analysis or because the data came from bioinformatics analysis platforms. Finally, a total of 9 studies (14–22) met the inclusion criteria and were included in this meta-analysis.

### Characteristics of included study

The main characteristics of the 9 included studies are presented in **Table 1**. All the studies originated from China and were published between 2018 and 2025. The sample size ranged from 42 to 134, involving a total of 938 tumor patients. Among them, 494 cases showed high DLEU1 expression, and 444 cases showed low DLEU1 expression. The tumor types mainly included cervical cancer, pancreatic ductal adenocarcinoma, nasopharyngeal carcinoma, gastric cancer, breast cancer, non-small cell lung cancer, osteosarcoma and hepatocellular carcinoma. All the included studies evaluated the relationship between DLEU1 expression level and overall survival, with follow-up periods ranging from 60 to 120 months. Eight studies assessed the relationship between DLEU1 expression level and clinicopathological parameters (age, gender, tumor differentiation, tumor size, lymph node metastasis and TNM stage). All the studies used qRT-PCR methods to detect the expression level of DLEU1. Furthermore, we conducted a quality assessment of the included studies using the NOS criteria. Each study achieved a NOS score of ≥ 5, indicating that they all met the standards of moderate to high quality.

**Table 1:** Characteristics of the included studies.

Study	Year	Region	Tumor type	Sample size (low/high)	HR (95% CI)	HR availability	Outcomes	DLEU1 Expression	Method	Follow-up months	NOS
Chen, Y (14).	2025	China	CC	134 (67/67)	0.47 (0.01, 16.97)	K-M curve	OS	Upregulated	qRT-PCR	66	7
Dong, A (15).	2022	China	CC	128 (62/66)	0.49 (0.18, 1.33)	K-M curve	OS, CP	Upregulated	qRT-PCR	60	7
Gao, S (16).	2019	China	PDAC	62 (23/39)	0.53 (0.31, 0.90)	K-M curve	OS, CP	Upregulated	qRT-PCR	60	7
Li, H. B (17).	2020	China	NPC	67 (20/47)	0.43 (0.21, 0.87)	K-M curve	OS, CP	Upregulated	qRT-PCR	120	6
Li, X (18).	2018	China	GC	68 (38/30)	0.32 (0.14, 0.71)	K-M curve	OS, CP	Upregulated	qRT-PCR	60	6
Wang, C (19).	2019	China	BC	60 (30/30)	0.50 (0.24, 1.05)	K-M curve	OS, CP	Upregulated	qRT-PCR	120	5
Zhang, J (20).	2018	China	NSCLC	42 (24/18)	0.56 (0.29, 1.09)	K-M curve	OS, CP	Upregulated	qRT-PCR	60	7
Zhang, J. J (21).	2023	China	OSC	86 (43/43)	0.58 (0.21, 1.61)	K-M curve	OS, CP	Upregulated	qRT-PCR	60	6
Zhang, W (22).	2019	China	HCC	56 (24/32)	0.49 (0.20, 1.23)	K-M curve	OS, CP	Upregulated	qRT-PCR	60	6

CC, cervical cancer; PDAC, pancreatic ductal adenocarcinoma; NPC, nasopharyngeal carcinoma; GC, gastric cancer; BC, breast cancer; NSCLC, non-small cell lung cancer; OSC, osteosarcoma; HCC, hepatocellular carcinoma; OS, overall survival; CP, clinicopathological parameters; HR, hazard ratios; CI, confidence intervals; NOS, Newcastle-Ottawa Scale.

### Association between the expression levels of DLEU1 and overall survival

**Figure 2:**
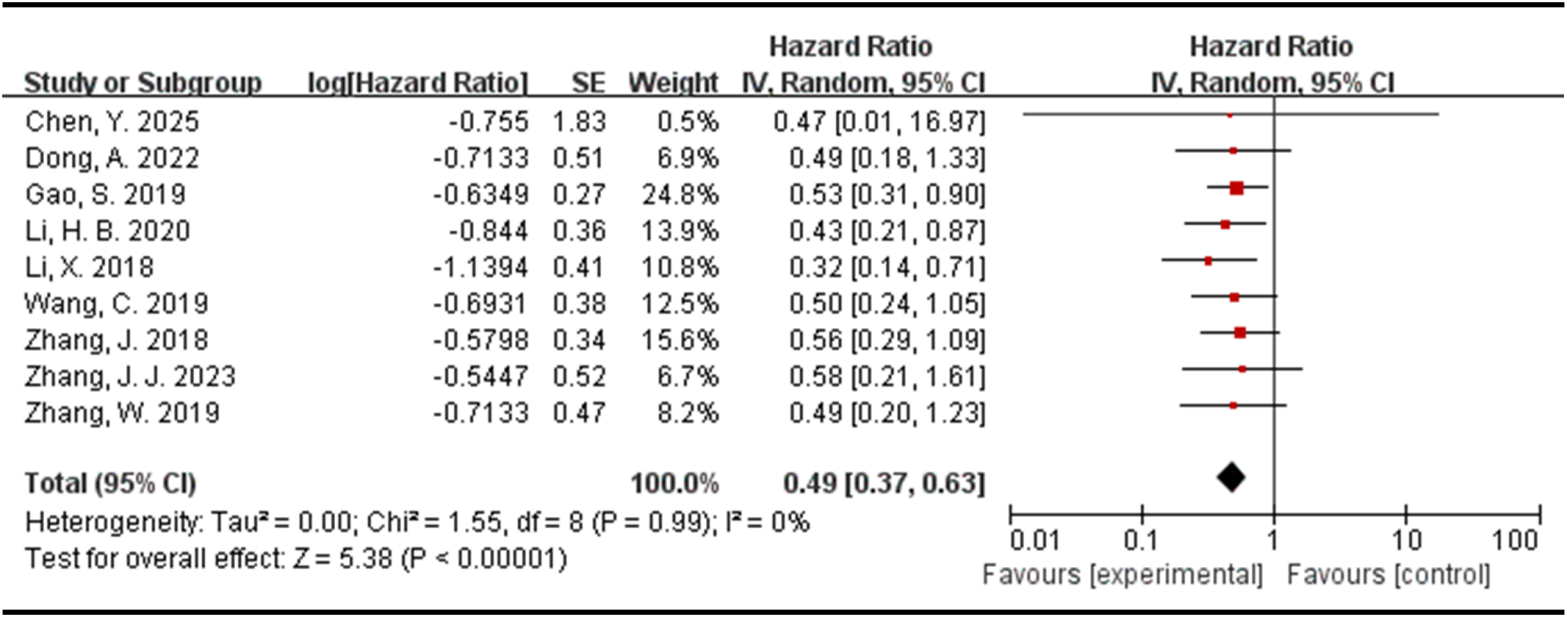
Forest plot for the association between DLEU1 expression levels and overall survival.

There were nine studies evaluated the association between the expression levels of DLEU1 and overall survival in this meta-analysis. As shown in **Figure 2**, random-effects model was used. The pooled HR = 0.49 (95% CI: 0.37-0.63, P<0.00001), which indicated that higher DLEU1 expression in the tumor tissues of patients with cervical cancer, pancreatic ductal adenocarcinoma, nasopharyngeal carcinoma, gastric cancer, breast cancer, non-small cell lung cancer, osteosarcoma and hepatocellular carcinoma were associated with a poor overall survival.

To assess the stability of the pooled outcome for overall survival, a sensitivity analysis was performed by sequentially omitting one study at a time. As shown in **Table 2**, after exclusion of any single study, the pooled HRs for OS ranged from 0.47 to 0.51, and all 95% confidence intervals remained below 1.0. The heterogeneity remained low throughout (I² = 0% in all iterations). These results indicate that no individual study disproportionately influenced the overall estimate, confirming the robustness of our findings.

**Table 2:** Sensitivity analysis: Pooled HRs for the association between DLEU1 expression and overall survival after omission of each study.

Study omitted	Pooled HR (95% CI)	Heterogeneity I² (%)
None (all studies included)	0.49 (0.37, 0.63)	0
Chen, Y. 2025 (14)	0.49 (0.37, 0.63)	0
Dong, A. 2022 (15)	0.48 (0.37, 0.64)	0
Gao, S. 2019 (16)	0.47 (0.35, 0.64)	0
Li, H. B. 2020 (17)	0.49 (0.37, 0.66)	0
Li, X. 2018 (18)	0.51 (0.39, 0.67)	0
Wang, C. 2019 (19)	0.48 (0.36, 0.64)	0
Zhang, J. 2018 (20)	0.47 (0.35, 0.63)	0
Zhang, J. J. 2023 (21)	0.48 (0.36, 0.63)	0
Zhang, W. 2019 (22)	0.48 (0.37, 0.64)	0

### Association between the expression levels of DLEU1 and tumor types

**Figure 3:**
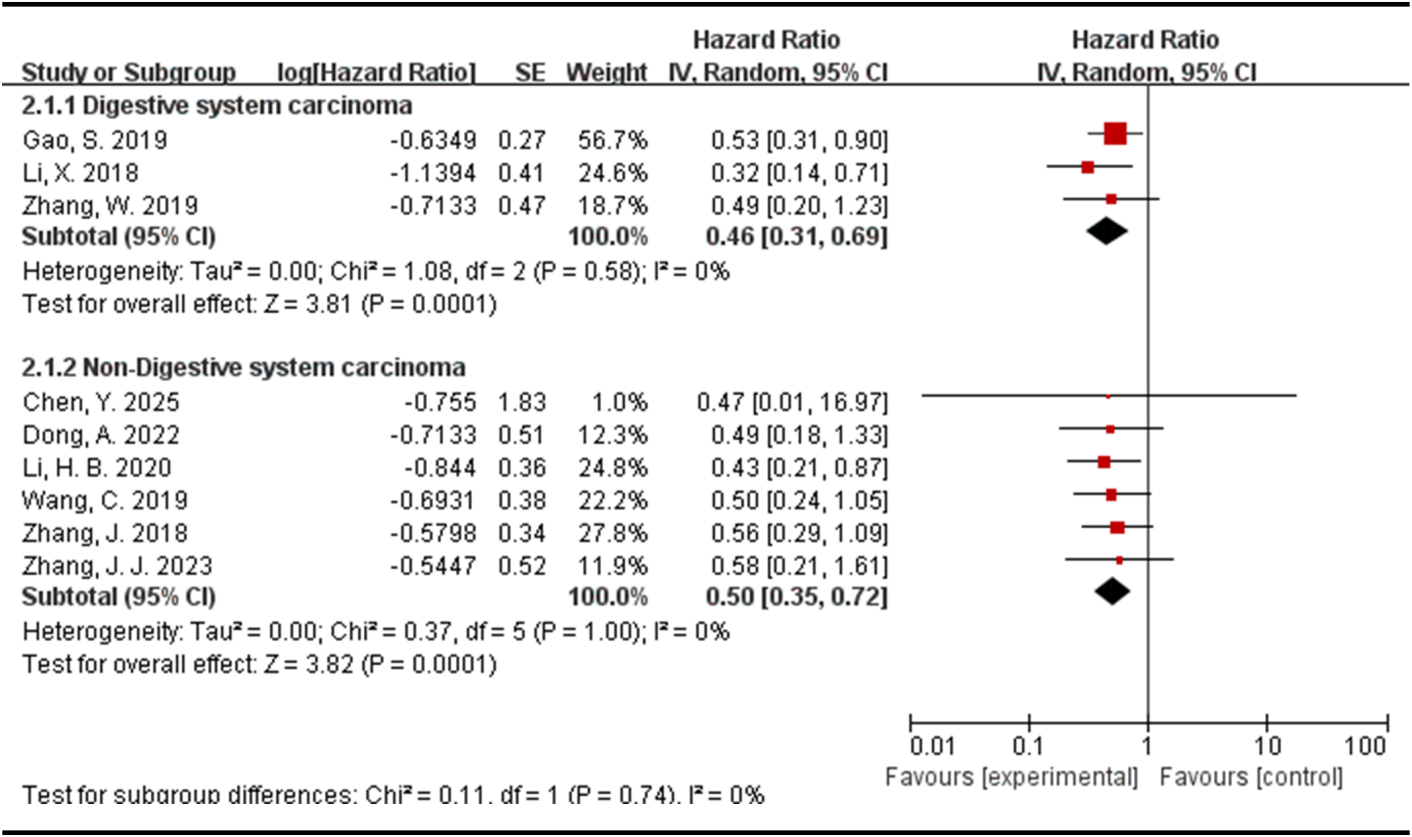
Forest plot for the association between DLEU1 expression levels and tumor types.

In order to further explore whether the prognostic value of DLEU1 is related to the tumor types, we classified and analyzed the 9 included studies according to digestive system carcinoma and non-digestive system carcinoma. After analyzing three studies related to digestive system carcinoma, it was found that there was no significant statistical heterogeneity among these studies (I^2^ = 0%, p=0.58). The random-effect model was used for statistical analysis, and the results showed that the pooled HR = 0.46 (95% CI: 0.31-0.69, p=0.0001, **Figure 3**). In the analysis of six studies related to non-digestive system carcinoma (I^2^ = 0%, p=1.00), a random-effect model was also used for statistical analysis, and the results showed that the pooled HR = 0.50 (95% CI: 0.35-0.72, p=0.0001, **Figure 3**). These results demonstrated that regardless of the digestive system carcinoma or non-digestive system carcinoma, the high expression of DLEU1 in tumor tissues was correlated with poor OS. Moreover, in the forest plot, the greater the distance between the diamond and the central axis, the more significant the analysis result. Since the pooled HR value of digestive system tumors is lower than that of non-digestive system tumors, it indicates that compared with non-digestive system tumors, the abnormal expression level of DLEU1 is more correlated with the OS of patients with digestive system tumors.

### Associations between the expression levels of DLEU1 and clinicopathological parameters

**Figure 4:**
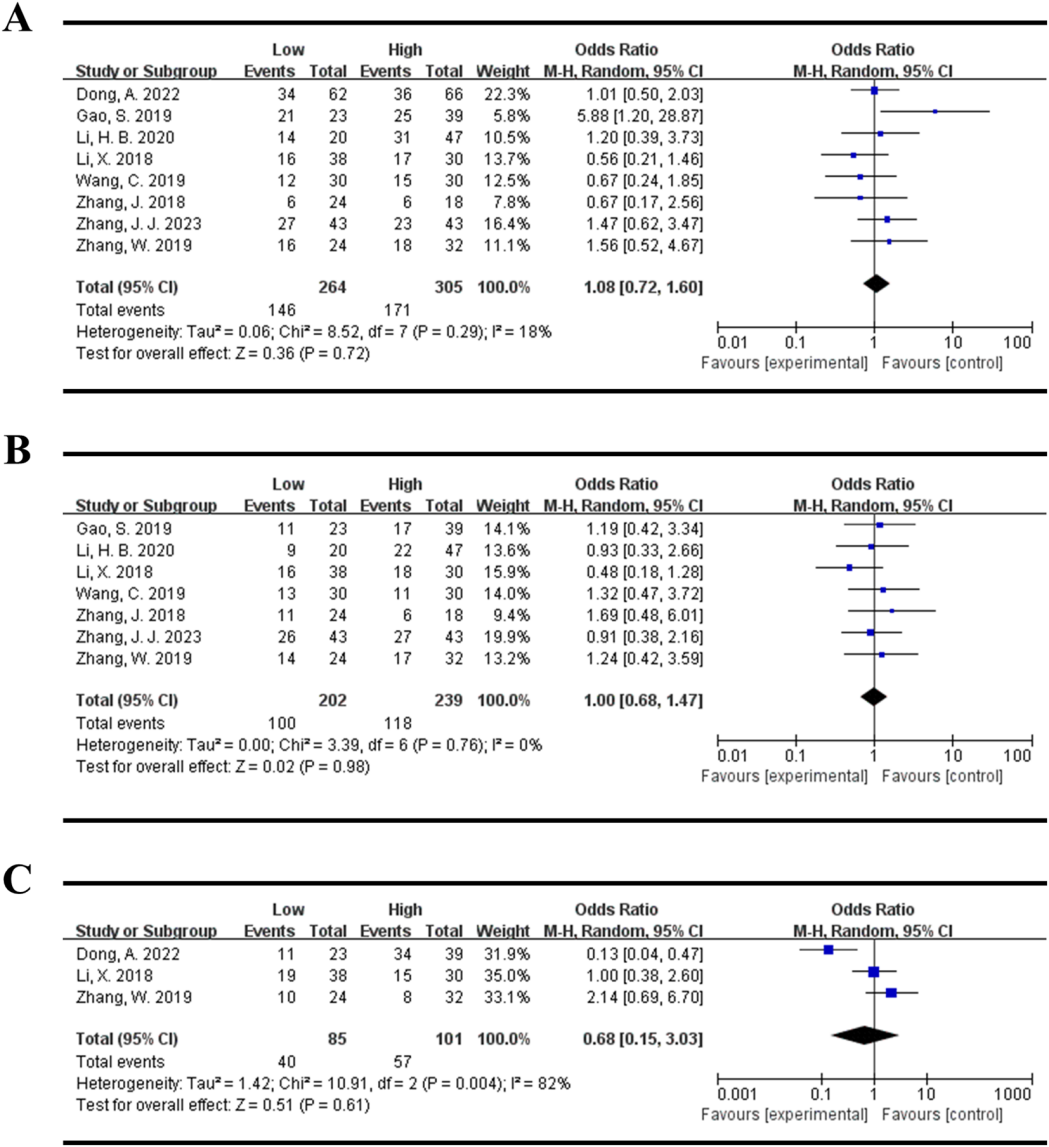
Forest plot for the association between DLEU1 expression levels and age **(A)**, gender **(B)**, tumor differentiation **(C)**.

**Figure 5:**
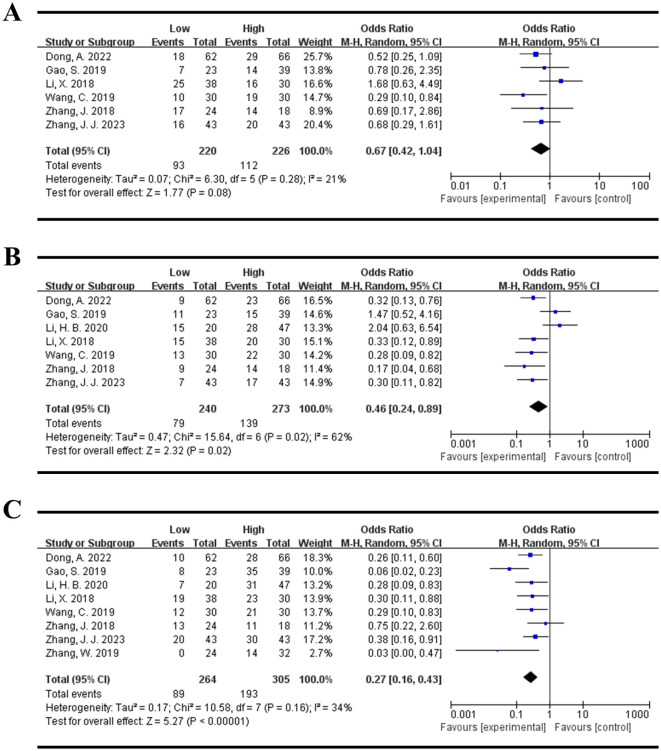
Forest plot for the association between DLEU1 expression levels and tumor size **(A)**, lymph node metastasis **(B)** TNM stage **(C)**.

There were 8 studies evaluated the association between the expression levels of DLEU1 and clinicopathological parameters in this meta-analysis. As shown in **Table 3**, there were no statistically significant relationships between high DLEU1 expression and age (total OR = 1.08, 95% CI:0.72-1.60, P = 0.72, Random model, **Figure 4A**), gender (total OR=1.00, 95% CI:0.68-1.47, P = 0.98, Random model, **Figure 4B**), tumor differentiation (total OR = 0.68, 95% CI:0.15-3.03, P = 0.61, Random model, **Figure 4C**) and tumor size (total OR = 0.67, 95% CI:0.42-1.04, P = 0.08, Random model, **Figure 5A**). However, high DLEU1 expression was statistically correlated with positive lymph node metastasis (OR = 0.46, 95% CI:0.24-0.89, P = 0.02, Random model, **Figure 5B**), and advanced TNM stage (OR = 0.27, 95% CI:0.16-0.43, P<0.00001, Random model, **Figure 5C**).

**Table 3:** Meta-analysis results for the association between DLEU1 expression and clinicopathological characteristics.

Characteristics	Studies (n)	Sample size	OR (95% CI)	*P* value	I^2^%	Model
Age (old vs young)	8	569	1.08 (0.72, 1.60)	0.72	18	Random
Gender (male vs female)	7	441	1.00 (0.68, 1.47)	0.98	0	Random
Differentiation (poor vs well)	3	186	0.68 (0.15, 3.03)	0.61	82	Random
Tumor size (large vs small)	6	446	0.67 (0.42, 1.04)	0.08	21	Random
Lymph node metastasis (yes vs no)	7	513	0.46 (0.24, 0.89)	0.02	62	Random
TNM stage (III-IV vs I-II)	8	569	0.27 (0.16, 0.43)	<0.00001	34	Random

OR, odds ratio; CI, confidence interval.

### Publication bias

**Figure 6:**
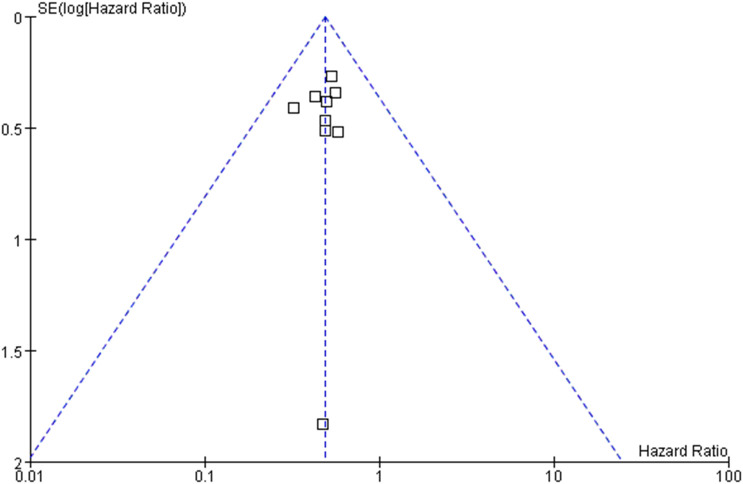
Funnel plots for the association between DLEU1 expression levels and overall survival.

The funnel plot was used to assess whether our research results were affected by potential publication bias. We analyzed the studies that reported the association between DLEU1 expression level and overall survival. As shown in **Figure 6**, the studies were evenly distributed on both sides of the central axis, indicating that there was no significant publication bias in these studies.

### Validation results of DLEU1 in public databases

**Figure 7:**
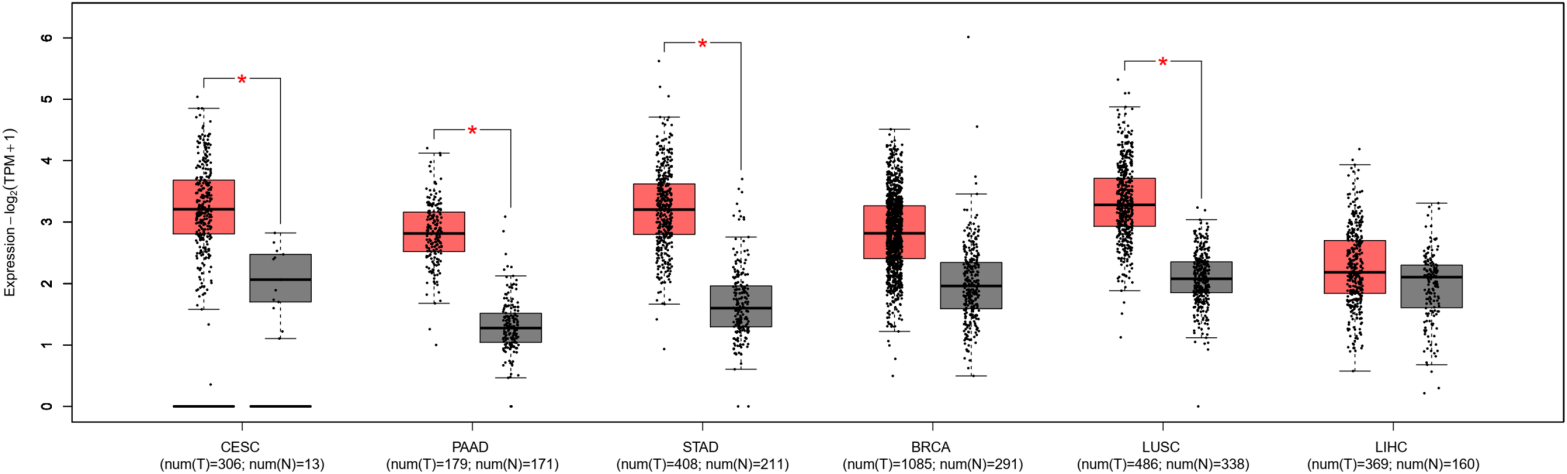
The expression levels of DLEU1 in six types of cancer tissues in GEPIA2 cohort. Red box plots, DLEU1 in cancer tissues; grey box plots, DLEU1 in normal tissues; *, p < 0.05.

**Figure 8:**
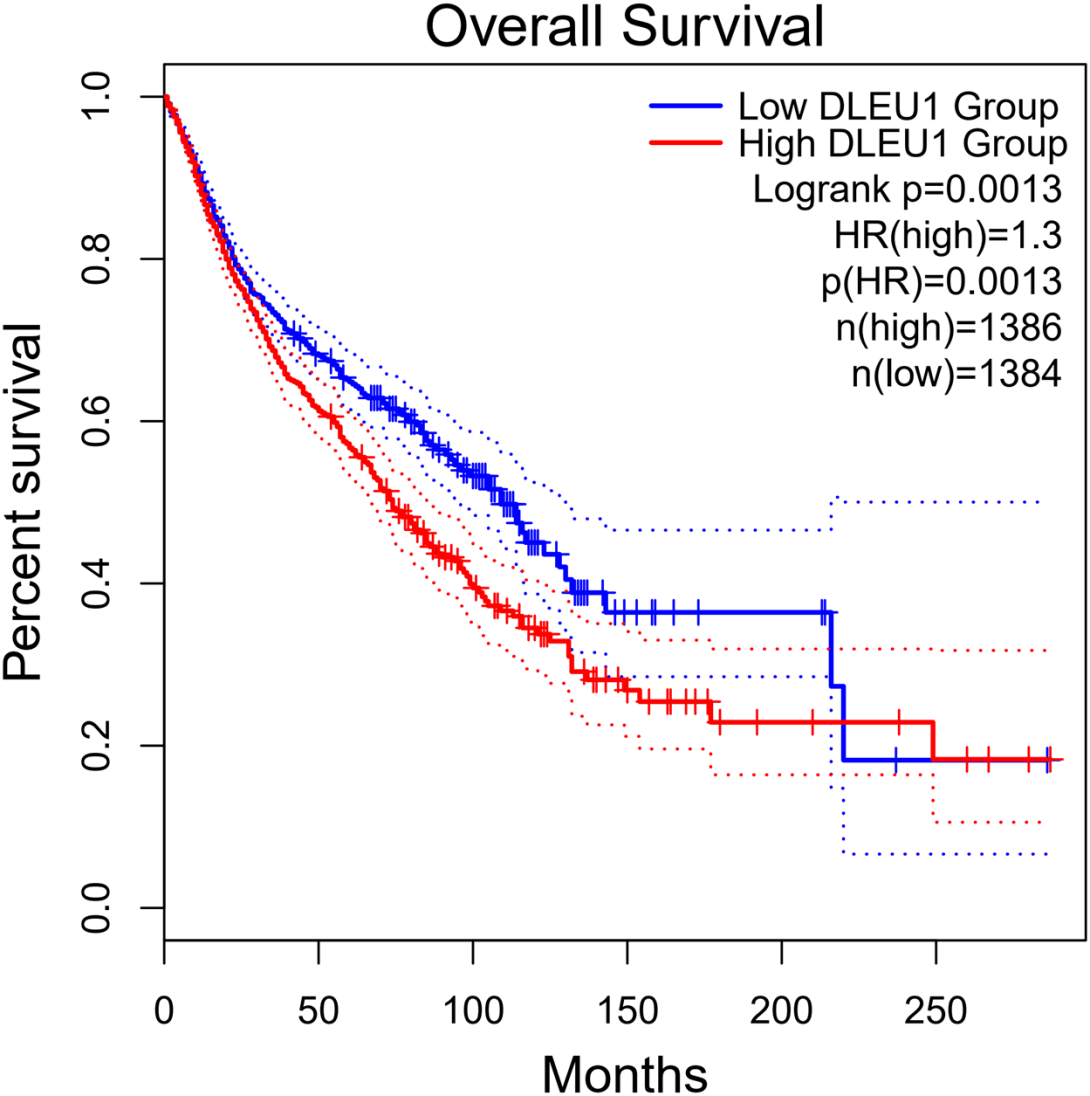
Overall survival plots of DLEU1 in GEPIA2 cohort, including CESC, PAAD, STAD, BRCA, LUSC and LIHC (n = 2770).

To further validate the results of this meta-analysis, we analyzed the expression levels of DLEU1 in eight types of cancers included in this study and its correlation with tumor prognosis based on the GEPIA 2 database. The results showed that compared with normal tissues, DLEU1 was significantly overexpressed in four types of tumor tissues, including cervical squamous cell carcinoma (CESC), pancreatic adenocarcinoma (PAAD), stomach adenocarcinoma (STAD) and lung squamous cell carcinoma (LUSC), and the difference was statistically significant (**Figure 7**, p<0.05). In breast invasive carcinoma (BRCA) and liver hepatocellular carcinoma (LIHC), although there was no significant statistical difference, the expression of DLEU1 also showed an upward trend (**Figure 7**, p>0.05). The expression levels of DLEU1 in nasopharyngeal carcinoma (NPC) and osteosarcoma (OSC) and their correlations with prognosis were not obtained in the GPIA2 database. In addition, we analyzed the correlation of DLEU1 with the prognosis of these six types of cancers (CESC, PAAD, STAD, BRCA, LUSC and LIHC). As shown in **Figure 8**, 2770 patients were divided into a high-expression group of DLEU1 (n = 1386) and a low-expression group (n = 1384), using the median expression level of DLEU1 in the six types of cancers as the cut-off value. The overall survival of the high-expression group of DLEU1 was lower than that of the low-expression group of DLEU1, which confirmed that high expression of DLEU1 in most human cancers is associated with poor prognosis.

### Prediction results of the functions and pathways of DLEU1

**Figure 9:**
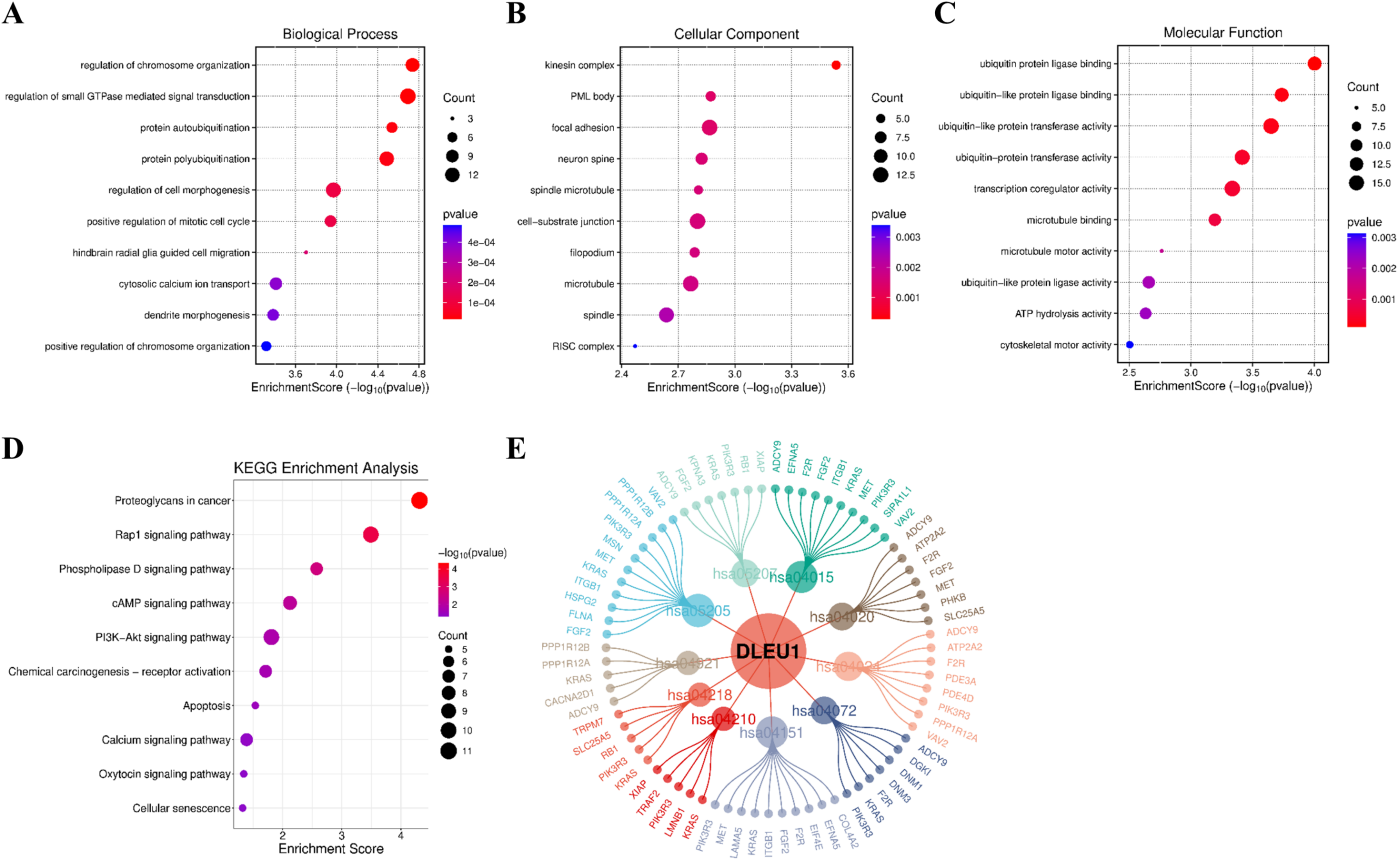
Bubble chart of GO enrichment of DLEU1-related target genes in biological processes **(A)**, cellular components **(B)** and molecular functions **(C)**; Bubble chart of KEGG enrichment analysis of DLEU1-related target genes **(D)**; The “DLEU1- signaling pathway-target genes” network diagram **(E)**.

To further clarify the functional mechanism of DLEU1, we predicted the potential target genes of DLEU1 based on the StarBase database, and then conducted GO and KEGG enrichment analyses on these target genes. A total of 254 target genes were obtained. The GO results showed that a total of 388 elements pertaining to biological processes (BP), 46 entries concerning cellular components (CC), and 55 elements relating to molecular functions (MF) were obtained. The BP primarily involved regulation of chromosome organization, positive regulation of mitotic cell cycle, and cytosolic calcium ion transport (**Figure 9A**; **Table 4**). The CC included the kinesin complex, focal adhesion, and microtubule (**Figure 9B**; **Table 4**). The MF primarily included p ubiquitin protein ligase binding, transcription coregulator activity, and microtubule motor activity (**Figure 9C**; **Table 4**). The KEGG enrichment analysis identified a total of 48 signaling pathways, mainly including the Rap1 signaling pathway, cAMP signaling pathway, PI3K-Akt signaling pathway, apoptosis and calcium signaling pathway (**Figure 9D**; **Table 5**). The “DLEU1- signaling pathway - target genes” network diagram was constructed and presented in **Figure 9E**. In future mechanism studies, experiments can be conducted on these signaling pathways to verify and reveal the molecular mechanism of DLEU1 in malignant tumors.

**Table 4:** Top 10 enrichment GO terms of the potential genes of DLEU1.

GO ID	Description	Ontology	Count	Pvalue
GO:0033044	regulation of chromosome organization	BP	11	1.83108E-05
GO:0051056	regulation of small GTPase mediated signal transduction	BP	14	2.02852E-05
GO:0051865	protein autoubiquitination	BP	7	2.89973E-05
GO:0000209	protein polyubiquitination	BP	12	3.26015E-05
GO:0022604	regulation of cell morphogenesis	BP	13	0.000107528
GO:0045931	positive regulation of mitotic cell cycle	BP	8	0.000114646
GO:0021932	hindbrain radial glia guided cell migration	BP	3	0.00019819
GO:0060401	cytosolic calcium ion transport	BP	9	0.000389498
GO:0048813	dendrite morphogenesis	BP	8	0.000414659
GO:2001252	positive regulation of chromosome organization	BP	6	0.000483158
GO:0005871	kinesin complex	CC	5	0.000291143
GO:0016605	PML body	CC	6	0.001344434
GO:0005925	focal adhesion	CC	13	0.001363032
GO:0044309	neuron spine	CC	8	0.001502111
GO:0005876	spindle microtubule	CC	5	0.001561248
GO:0030055	cell-substrate junction	CC	13	0.001579722
GO:0030175	filopodium	CC	6	0.001634951
GO:0005874	microtubule	CC	13	0.001715886
GO:0005819	spindle	CC	12	0.002306234
GO:0016442	RISC complex	CC	3	0.003380112
GO:0031625	ubiquitin protein ligase binding	MF	13	9.97205E-05
GO:0044389	ubiquitin-like protein ligase binding	MF	13	0.000184252
GO:0019787	ubiquitin-like protein transferase activity	MF	16	0.000224617
GO:0004842	ubiquitin-protein transferase activity	MF	15	0.000384899
GO:0003712	transcription coregulator activity	MF	16	0.000462878
GO:0008017	microtubule binding	MF	11	0.000641397
GO:0003777	microtubule motor activity	MF	5	0.001727326
GO:0061659	ubiquitin-like protein ligase activity	MF	11	0.002197709
GO:0016887	ATP hydrolysis activity	MF	10	0.002332507
GO:0003774	cytoskeletal motor activity	MF	6	0.003132101

BP, biological processes; CC, cellular components; MF, molecular functions.

**Table 5:** KEGG pathway enrichment analysis of the potential target genes of DLEU1.

ID	Description	GeneID	Count	Pvalue
hsa05205	Proteoglycans in cancer	PPP1R12A, PPP1R12B, PIK3R3, KRAS, FGF2, ITGB1, MET, HSPG2, MSN, VAV2, FLNA	11	4.85119E-05
hsa04015	Rap1 signaling pathway	PIK3R3, KRAS, FGF2, ITGB1, ADCY9, MET, VAV2, EFNA5, SIPA1L1, F2R	10	0.000322805
hsa04072	Phospholipase D signaling pathway	PIK3R3, KRAS, DGKI, ADCY9, DNM1, DNM3, F2R	7	0.002662949
hsa04024	cAMP signaling pathway	PPP1R12A, PDE4D, PIK3R3, ADCY9, ATP2A2, VAV2, PDE3A, F2R	8	0.007507035
hsa04151	PI3K-Akt signaling pathway	PIK3R3, KRAS, COL4A2, FGF2, ITGB1, MET, LAMA5, EIF4E, EFNA5, F2R	10	0.015500364
hsa05207	Chemical carcinogenesis receptor activation	KPNA3, PIK3R3, KRAS, FGF2, RB1, ADCY9, XIAP	7	0.019352226
hsa04210	Apoptosis	PIK3R3, KRAS, XIAP, LMNB1, TRAF2	5	0.028926482
hsa04020	Calcium signaling pathway	SLC25A5, FGF2, ADCY9, ATP2A2, PHKB, MET, F2R	7	0.040656433
hsa04921	Oxytocin signaling pathway	PPP1R12A, PPP1R12B, KRAS, CACNA2D1, ADCY9	5	0.045408597
hsa04218	Cellular senescence	SLC25A5, TRPM7, PIK3R3, KRAS, RB1	5	0.047525662

### Summary of the biological functions and mechanisms of DLEU1 in cancers

**Figure 10:**
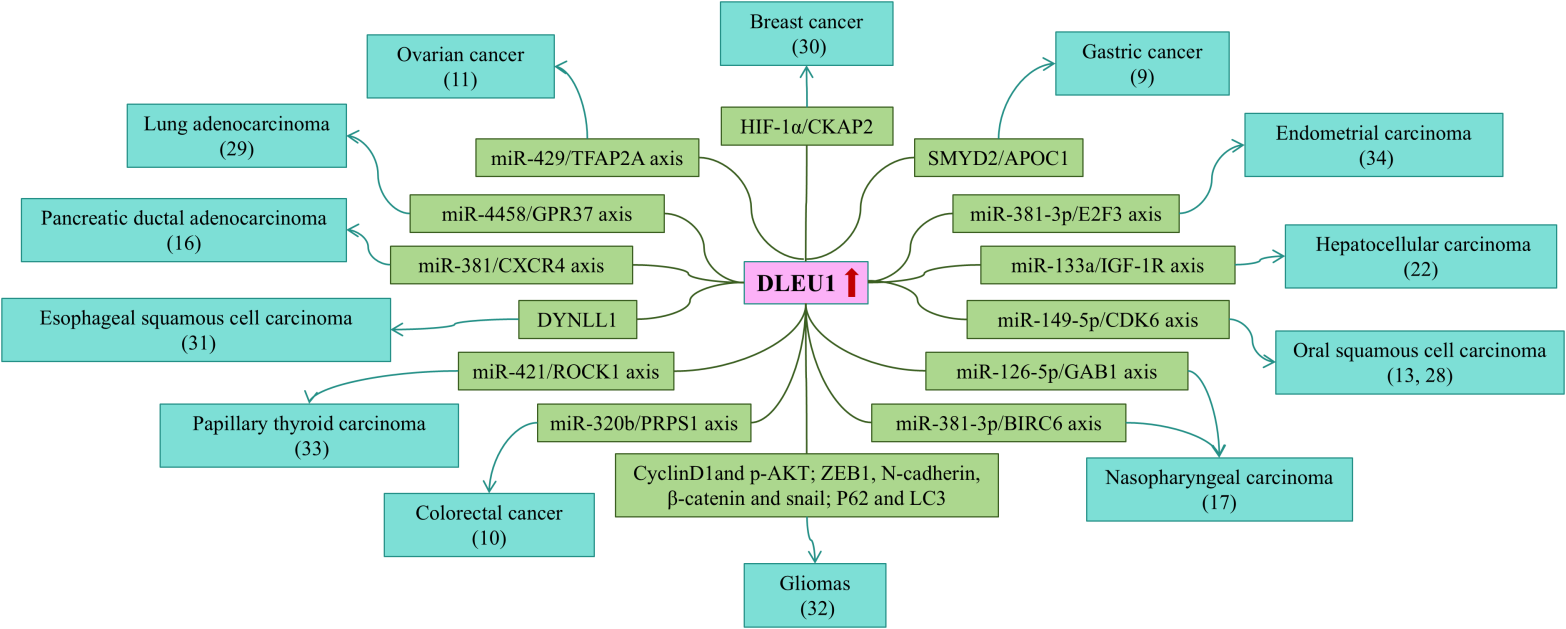
. A network diagram showing the correlations between DLEU1 expression levels, signaling pathways, and cancer types.

To elucidate the role of DLEU1 across various cancers, we reviewed literature from the past five years focusing on its biological functions and molecular mechanisms. A comprehensive summary is provided in **Table 6**; **Figure 10**.

**Table 6:** Summary of the biological functions and mechanisms of DLEU1 in various cancers.

Cancer types	Expression	Pathways/target genes	Biological function	Role	Reference
Gastric cancer	Upregulated	SMYD2/APOC1	Promotes cell proliferation and glycolysis	Oncogene	(9)
Colorectal cancer	Upregulated	miR-320b/PRPS1 axis	Promotes cell proliferation, migration, and invasion; Inhibits cell apoptosis	Oncogene	(10)
Ovarian cancer	Upregulated	miR-429/TFAP2A axis	Promotes cell proliferation, migration, and invasion	Oncogene	(11)
Oral squamous cell carcinoma	Upregulated	miR−149−5p/CDK6 axis	Promotes cell proliferation, migration, and invasion; Inhibits cell apoptosis	Oncogene	(13)
Nasopharyngeal carcinoma	Upregulated	miR-381-3p/BIRC6 axis	promote cisplatin resistance	Oncogene	(17)
Oral squamous cell carcinoma	Upregulated	miR‐126‐5p/GAB1 axis	Promotes cell proliferation, migration, and invasion	Oncogene	(28)
Lung adenocarcinoma	Upregulated	miR-4458/GPR37 axis	Promotes cell proliferation, migration, and invasion	Oncogene	(29)
Breast cancer	Upregulated	HIF-1α/CKAP2	Promotes cell proliferation, migration, and invasion	Oncogene	(30)
Esophageal squamous cell carcinoma	Upregulated	DYNLL1	Promotes cell proliferation, migration, and invasion; Inhibits cell apoptosis	Oncogene	(31)
Gliomas	Upregulated	CyclinD1and p-AKT; ZEB1, N-cadherin, β-catenin and snail; P62 and LC3	Promotes cell proliferation, migration, and invasion; Inhibits cell apoptosis and temozolomide chemosensitivity	Oncogene	(32)
Papillary thyroid carcinoma	Upregulated	miR-421/ROCK1 axis	Promotes cell proliferation, migration, and invasion; Inhibits cell apoptosis	Oncogene	(33)
Endometrial carcinoma	Upregulated	miR-381-3p/E2F3 axis	Promotes cell proliferation, migration, and invasion	Oncogene	(34)

In colorectal cancer, DLEU1 is markedly upregulated. Knockdown of DLEU1 inhibited proliferation, migration, and invasion in LoVo and SW480 cells, while promoting apoptosis, through suppression of phosphoribosyl pyrophosphate synthetase 1 (PRPS1) expression via the miR-320b pathway (10).

In ovarian cancer, DLEU1 is significantly overexpressed. Its silencing impeded proliferation, migration, and invasion of cancer cells by modulating the miR-429/transcription factor AP-2 alpha (TFAP2A) axis (11).

In oral squamous cell carcinoma, DLEU1 is notably upregulated. Knockdown of DLEU1 inhibited proliferation, migration, and invasion, and induced apoptosis in Cal-27 cells via the miR-149-5p/CDK6 axis (13). Another study demonstrated that silencing DLEU1 suppressed the proliferation, migration, and invasion of CAL-27 and SCC-25 cells, whereas its overexpression conversely promoted these malignant phenotypes. This effect was mechanistically linked to the regulation of the miR-126-5p/Grb2-associated binder 1 (GAB1) axis (28).

In nasopharyngeal carcinoma tissues, DLEU1 is significantly upregulated and function as a ceRNA to sponge miR-381-3p, thereby positively regulating baculoviral IAP repeat containing protein 6 (BIRC6) expression. Silencing DLEU1 enhanced the sensitivity of cancer cells to DDP treatment both *in vitro* and *in vivo* (17).

In lung adenocarcinoma, DLEU1 is significantly upregulated. By functioning as a molecular sponge for miR-4458, DLEU1 modulates the expression of its downstream target G protein-coupled receptor 37 (GPR37), ultimately enhancing the proliferative, migratory and invasive capacities of Calu-3 and A549 cells (29).

In gastric cancer, DLEU1 is upregulated, where it facilitates the recruitment of SET and MYND Domain-Containing Protein 2 (SMYD2) to upregulate ApolipoproteinC1 (APOC1), ultimately enhancing proliferation and glycolysis in MKN45 and SGC7901 cells (9).

In breast cancer, DLEU1 expression is significantly upregulated. Silencing DLEU1 effectively inhibited the proliferation and migration of MDA-MB-468 and MCF7 cells. Mechanistically, DLEU1 interacts with hypoxia-inducible factor 1α (HIF-1α) to cooperatively activate the transcription of cytoskeleton-associated protein 2 (CKAP2) (30).

In esophageal squamous cell carcinoma, DLEU1 is significantly overexpressed and mediates cancer cell proliferation, migration, invasion, and apoptosis through regulation of Dynein light chain LC8-type 1 (DYNLL1) (31).

In glioma, DLEU1 expression is significantly elevated compared to normal brain tissue. Knockdown of DLEU1 inhibited cell proliferation by inducing cell cycle arrest through downregulation of cyclin D1 and phosphorylated AKT. Concurrently, it suppressed cell migration and invasion by inhibiting epithelial-mesenchymal transition (EMT) markers, including ZEB1, N-cadherin, β-catenin, and Snail. Furthermore, DLEU1 silencing attenuated TMZ-induced autophagy by modulating P62 and LC3 protein expression and promoted apoptosis, thereby potentiating glioma cell sensitivity to temozolomide (32).

In papillary thyroid carcinoma, DLEU1 is highly expressed and promotes tumor progression by sponging miR-421 and increasing rho-related coiled-coil kinase 1 (ROCK1) expression (33).

In endometrial cancer, DLEU1 is significantly upregulated and facilitates proliferation, migration, and invasion through regulation of the miR-381-3p/E2F transcription factor 3 (E2F3) axis (34).

## Discussion

Cancer remains one of the leading causes of death worldwide, posing a substantial threat to human health. The exploration of tumor biomarkers for early detection and intervention is therefore of critical importance. Advances in high-throughput technologies have unveiled the critical roles of long non-coding RNAs (lncRNAs) in tumor biology, establishing them as molecules of significant prognostic and functional relevance. In recent years, several lncRNAs such as SNHG5 (35), HOTAIR (36), and MALAT1 (37) have been demonstrated to correlate closely with survival outcomes and clinicopathological features in cancer patients. Moreover, dysregulated lncRNAs can function as either tumor suppressors or oncogenes, thereby mediating tumor initiation and progression. Consequently, lncRNAs have emerged as a promising class of prognostic biomarkers and therapeutic targets in cancer.

DLEU1, a cancer-associated long non-coding RNA, has been demonstrated to be significantly overexpressed in multiple malignancies and is regarded as a novel prognostic biomarker. For instance, Song Gao et al. revealed that DLEU1 was markedly upregulated in pancreatic ductal adenocarcinoma (PDAC) tissues compared with adjacent normal tissues. Moreover, its high expression was correlated with poor differentiation, lymph node metastasis, and reduced survival in PDAC patients (16). Similarly, Xiaobin Li et al. reported elevated DLEU1 expression in human gastric cancer tissues and cell lines, where increased DLEU1 levels were closely associated with lymph node metastasis, advanced TNM stage, and poorer overall survival (18). In addition, several other studies have confirmed that aberrant DLEU1 expression is closely linked to clinicopathological features and prognosis in various cancers (17, 19–22). However, due to limitations such as small sample sizes in individual studies, the prognostic value of DLEU1 remains controversial. To address this issue, we conducted this meta-analysis to systematically evaluate the correlation between dysregulated DLEU1 expression and both prognosis and clinicopathological characteristics in cancer patients.

This study represents the first meta-analysis to comprehensively evaluate the association between dysregulated DLEU1 expression and both prognosis and clinicopathological parameters in cancer patients. Our results demonstrate that elevated DLEU1 expression is significantly associated with poorer survival across multiple malignancies, including cervical cancer, pancreatic ductal adenocarcinoma, nasopharyngeal carcinoma, gastric cancer, breast cancer, non-small cell lung cancer, osteosarcoma, and hepatocellular carcinoma. Subgroup analysis revealed that the prognostic value of DLEU1 was more pronounced in digestive system cancers (pooled HR = 0.46) than in non-digestive cancers (HR = 0.50). Among these, gastric cancer and pancreatic ductal adenocarcinoma showed the strongest associations with poor survival, suggesting their potential as priority candidates for DLEU1-based diagnostic biomarker development. No statistically significant associations were observed between DLEU1 overexpression and patient age, gender, tumor differentiation grade or tumor size. However, high DLEU1 expression showed a statistically significant correlation with positive lymph node metastasis, and advanced TNM stage. To further validate these findings, DLEU1 expression patterns and survival correlations were examined using the GEPIA2 database. The results confirmed significantly upregulated DLEU1 expression in four cancer types (cervical squamous cell carcinoma, pancreatic adenocarcinoma, stomach adenocarcinoma, and lung squamous cell carcinoma), compared to normal tissues. In breast invasive carcinoma and liver hepatocellular carcinoma, although not statistically significant, an upward trend in DLEU1 expression was noted (p > 0.05). Survival analysis further revealed that patients with low DLEU1 expression exhibited prolonged overall survival compared to those with high expression, consistent with our meta-analysis results.

In summary, dysregulated DLEU1 expression is closely associated with cancer prognosis and may serve as a promising biomarker across multiple malignancies. To explore its functional mechanisms, we predicted potential target genes of DLEU1 using the StarBase database and performed GO and KEGG enrichment analyses. A total of 254 target genes of DLEU1 were identified, and these target genes were significantly enriched in several cancer-related pathways, including the Rap1 signaling pathway, cAMP signaling pathway, PI3K-Akt signaling pathway, apoptosis, and calcium signaling pathway. While DLEU1 frequently functions as a miRNA sponge in a ceRNA network, our systematic review (summarized in **Table 6**) also highlights its versatility in regulating tumorigenesis through diverse, miRNA-independent mechanisms. These include direct protein interactions, epigenetic regulation, and transcriptional coactivation, underscoring its complex role as a multi-faceted oncogene. According to the published literature, DLEU1 is significantly overexpressed in hepatocellular carcinoma tissues and cell lines, where it functions as an oncogene to promote malignant phenotypes. Silencing DLEU1 markedly reduces the activity of the PI3K/AKT signaling pathway (22). These findings not only clarify the functional relationship between DLEU1 and the PI3K/AKT signaling pathway but also validate the reliability of our bioinformatic predictions. In addition to the PI3K/AKT signaling pathway, other predicted signaling pathways provide valuable references for future mechanistic studies targeting DLEU1.

While our study elucidates the prognostic value of DLEU1 in cancer and summarizes its oncogenic mechanisms, several limitations should be acknowledged. First, although our search strategy imposed no language or geographic restrictions, all eligible studies ultimately originated from China. This geographic concentration may introduce selection bias and limit the generalizability of our findings to other ethnic populations. Future studies with diverse geographic cohorts are warranted to validate the prognostic role of DLEU1 globally. Furthermore, factors such as limited sample sizes, varying cancer types, and differences in follow-up durations may contribute to the heterogeneity observed in this meta-analysis. Although only nine studies were included, this analysis still provides valuable preliminary insights into the potential role of DLEU1 in cancer prognosis. We recommend that future research incorporate larger sample sizes and higher-quality studies to further validate our findings and enhance reliability. Second, hazard ratios and corresponding 95% confidence intervals were indirectly estimated by reconstructing survival curves using the Engage Digitizer software. While this approach does not alter the overall trends of the meta-analysis results, it may reduce data precision. Additionally, bioinformatics analyses predicted several signaling pathways as potential mechanisms through which DLEU1 regulates tumor development. Although these findings offer valuable references for future basic research, further *in vitro* and *in vivo* experiments are necessary for validation.

Our findings support the potential incorporation of DLEU1 into prognostic risk stratification models, particularly for digestive system cancers. As evidence accumulates, lncRNA-based biomarkers like DLEU1 may inform future updates of clinical practice guidelines for personalized oncology.

## Conclusion

In summary, the aberrant expression of lncRNA DLEU1 is significantly associated with adverse clinicopathological features and unfavorable prognosis in patients with various cancers. DLEU1 may serve as a novel and promising prognostic biomarker and therapeutic target for multiple human cancers. However, our study has several limitations, and future research with high-quality, large-scale, and multi-center cohorts is required to validate these findings.

## Data Availability

The original contributions presented in the study are included in the article/supplementary material. Further inquiries can be directed to the corresponding author.
